# ElliPro: a new structure-based tool for the prediction of antibody epitopes

**DOI:** 10.1186/1471-2105-9-514

**Published:** 2008-12-02

**Authors:** Julia Ponomarenko, Huynh-Hoa Bui, Wei Li, Nicholas Fusseder, Philip E Bourne, Alessandro Sette, Bjoern Peters

**Affiliations:** 1 San Diego Supercomputer Center, University of California, San Diego, 9500 Gilman Drive, La Jolla, California 92093, USA; 2 Skaggs School of Pharmacy and Pharmaceutical Sciences, University of California, San Diego, 9500 Gilman Drive, La Jolla, California 92093, USA; 3 Isis Pharmaceuticals, Inc., 1896 Rutherford Road, Carlsbad, California 92008, USA; 4 La Jolla Institute for Allergy and Immunology, 9420 Athena Circle, La Jolla, California 92037, USA

## Abstract

**Background:**

Reliable prediction of antibody, or B-cell, epitopes remains challenging yet highly desirable for the design of vaccines and immunodiagnostics. A correlation between antigenicity, solvent accessibility, and flexibility in proteins was demonstrated. Subsequently, Thornton and colleagues proposed a method for identifying continuous epitopes in the protein regions protruding from the protein's globular surface. The aim of this work was to implement that method as a web-tool and evaluate its performance on discontinuous epitopes known from the structures of antibody-protein complexes.

**Results:**

Here we present ElliPro, a web-tool that implements Thornton's method and, together with a residue clustering algorithm, the MODELLER program and the Jmol viewer, allows the prediction and visualization of antibody epitopes in a given protein sequence or structure. ElliPro has been tested on a benchmark dataset of discontinuous epitopes inferred from 3D structures of antibody-protein complexes. In comparison with six other structure-based methods that can be used for epitope prediction, ElliPro performed the best and gave an AUC value of 0.732, when the most significant prediction was considered for each protein. Since the rank of the best prediction was at most in the top three for more than 70% of proteins and never exceeded five, ElliPro is considered a useful research tool for identifying antibody epitopes in protein antigens. ElliPro is available at .

**Conclusion:**

The results from ElliPro suggest that further research on antibody epitopes considering more features that discriminate epitopes from non-epitopes may further improve predictions. As ElliPro is based on the geometrical properties of protein structure and does not require training, it might be more generally applied for predicting different types of protein-protein interactions.

## Background

An antibody epitope, aka B-cell epitope or antigenic determinant, is a part of an antigen recognized by either a particular antibody molecule or a particular B-cell receptor of the immune system [1]. For a protein antigen, an epitope may be either a short peptide from the protein sequence, called a continuous epitope, or a patch of atoms on the protein surface, called a discontinuous epitope. While continuous epitopes can be directly used for the design of vaccines and immunodiagnostics, the objective of discontinuous epitope prediction is to design a molecule that can mimic the structure and immunogenic properties of an epitope and replace it either in the process of antibody production–in this case an epitope mimic can be considered as a prophylactic or therapeutic vaccine–or antibody detection in medical diagnostics or experimental research [2,3].

If continuous epitopes can be predicted using sequence-dependent methods built on available collections of immunogenic peptides (for review see [4]), discontinuous epitopes–that are mostly the case when a whole protein, pathogenic virus, or bacteria is recognized by the immune system–are difficult to predict or identify from functional assays without knowledge of a three-dimensional (3D) structure of a protein [5,6]. The first attempts at epitope prediction based on 3D protein structure began in 1984 when a correlation was established between crystallographic temperature factors and several known continuous epitopes of tobacco mosaic virus protein, myoglobin and lysozyme [7]. A correlation between antigenicity, solvent accessibility, and flexibility of antigenic regions in proteins was also found [8]. Thornton and colleagues [9] proposed a method for identifying continuous epitopes in the protein regions protruding from the protein's globular surface. Regions with high protrusion index values were shown to correspond to the experimentally determined continuous epitopes in myoglobin, lysozyme and myohaemerythrin [9].

Here we present ElliPro (derived from Ellipsoid and Protrusion), a web-tool that implements a modified version of Thornton's method [9] and, together with a residue clustering algorithm, the MODELLER program [10] and the Jmol viewer, allows the prediction and visualization of antibody epitopes in protein sequences and structures. ElliPro has been tested on a benchmark dataset of epitopes inferred from 3D structures of antibody-protein complexes [11] and compared with six structure-based methods, including the only two existing methods developed specifically for epitope prediction, CEP [12] and DiscoTope [13]; two protein-protein docking methods, DOT [14] and PatchDock [15]; and two structure-based methods for protein-protein binding site prediction, PPI-PRED [16] and ProMate [17]. ElliPro is available at .

## Implementation

### The tool input

**Figure 1:**
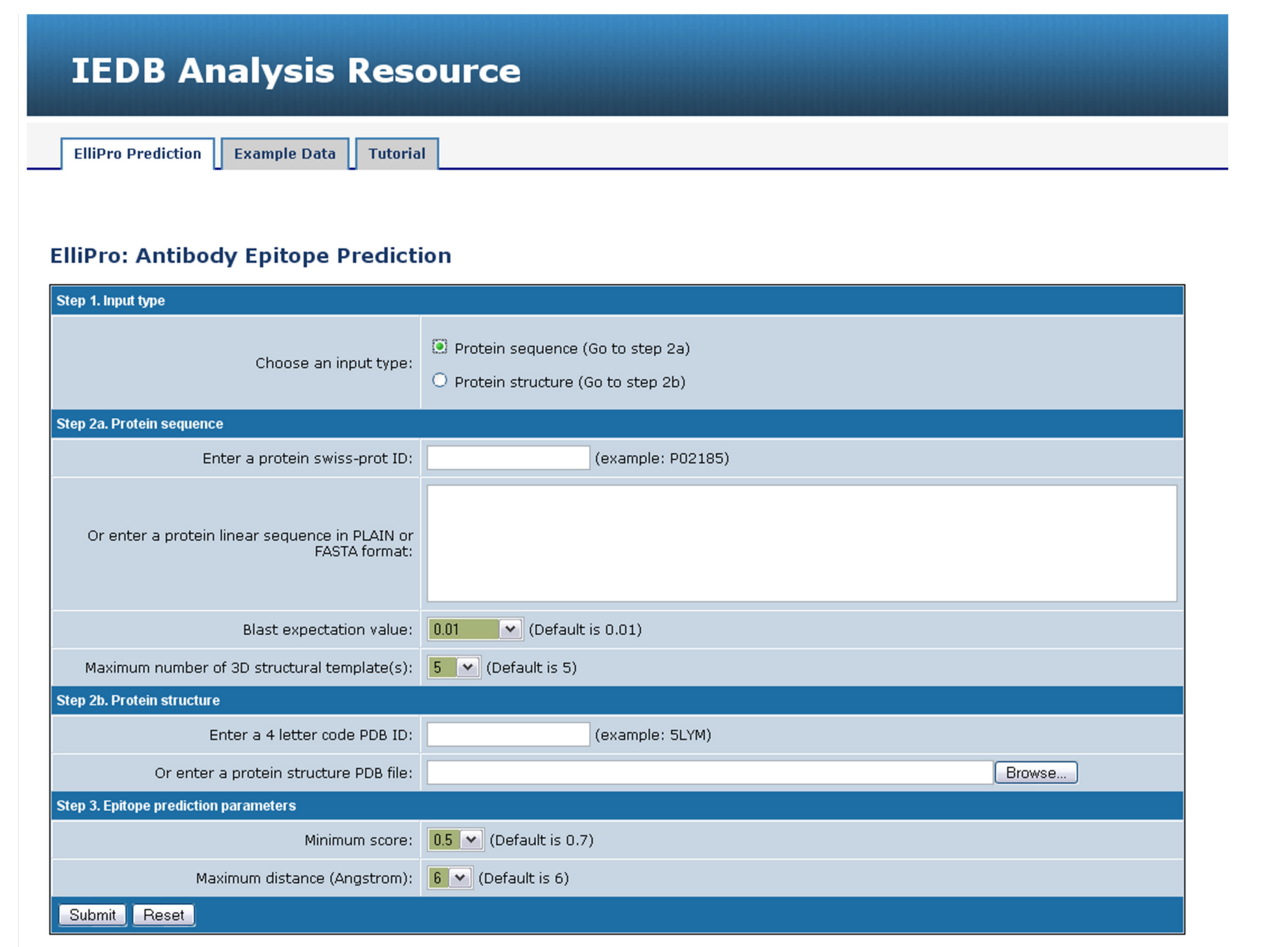
Screen shot of ElliPro input page.

ElliPro is implemented as a web accessible application and accepts two types of input data: protein sequence or structure (Fig. 1, Step 1). In the first case, the user may input either a protein SwissProt/UniProt ID or a sequence in either FASTA format or single letter codes and select threshold values for BLAST e-value and the number of structural templates from PDB that will be used to model a 3D structure of the submitted sequence (Fig. 1, Step 2a). In the second case, the user may input either a four-character PDB ID or submit her own PDB file in PDB format (Fig. 1, Step 2b). If the submitted structure consists of more than one protein chain, ElliPro will ask the user to select the chain(s) upon which to base the calculation. The user can change threshold values on the parameters used by ElliPro for epitope prediction, namely, the minimum residue score (protrusion index), denoted here as *S*, between 0.5 and 1.0 and the maximum distance, denoted as *R*, in the range 4 – 8Å.

### 3D Structure Modeling

If a protein sequence is used as input, ElliPro searches for the protein or its homologues in PDB [18], using a BLAST search [19]. If a protein cannot be found in PDB that matches the BLAST criteria, MODELLER [10] is run to predict the protein 3D structure. The user may change the threshold values for BLAST e-value and a number of templates that MODELLER uses as an input (Fig. 1, Step 2a).

### ElliPro Method

ElliPro implements three algorithms performing the following tasks: (i) approximation of the protein shape as an ellipsoid [20]; (ii) calculation of the residue protrusion index (PI) [9]; and (iii) clustering of neighboring residues based on their PI values.

Thornton's method for continuous epitope prediction was based on the two first algorithms and only considered Cα atoms [9]. It approximated the protein surface as an ellipsoid, which can vary in sizes to include different percentages of the protein atoms; for example, the 90% ellipsoid includes 90% of the protein atoms. For each residue, a protrusion index (PI) was defined as percentage of the protein atoms enclosed in the ellipsoid at which the residue first becomes lying outside the ellipsoid; for example, all residues that are outside the 90% ellipsoid will have PI = 9 (or 0.9 in ElliPro). In implementing the first two algorithms, ElliPro differs from Thornton's method by considering each residue's center of mass rather than its Cα atom.

The third algorithm for clustering residues defines a discontinuous epitope based on the threshold values for the protrusion index *S *and the distance *R *between each residue's centers of mass. All protein residues with a PI values greater than *S *are considered when calculating discontinuous epitopes. Clustering separate residues into discontinuous epitopes involves three steps that are recursively repeated until distinct clusters with no overlapping residues are formed. First, primary clusters are formed from single residues and their neighboring residues within the distance *R*. Second, secondary clusters are formed from primary clusters where at least three centers of mass are within the distance *R *from each other. Third, tertiary clusters are formed from secondary clusters which contain common residues. These tertiary clusters of residues represent distinct discontinuous epitopes predicted in the protein. The score for each epitope is defined as a PI value averaged over epitope residues.

### 3D visualization of Predicted Epitopes

**Figure 2:**
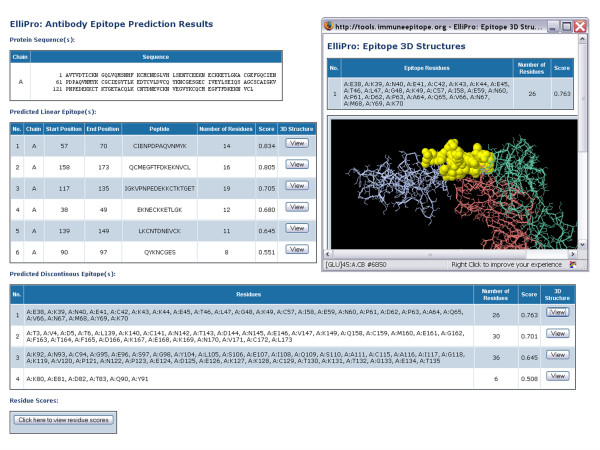
**Screen shots of the ElliPro result page for *Plasmodium vivax *ookinete surface protein Pvs25 [PDB:**1Z3G, **chain A] and Jmol visualization of the first of the four predicted epitopes**. The epitope residues are in yellow, the rest of the protein is in violet, antibody chains are in green and brown.

An open-source molecular viewer Jmol [21] was used to visualize linear and discontinuous epitopes on the protein 3D structure. An example of epitope visualization is shown in Fig. 2.

## Results and Discussion

For evaluation of ElliPro performance and comparison with other methods we used a previously established benchmark approach for discontinuous epitopes [11]. We tested ElliPro on a dataset of 39 epitopes present in 39 protein structures where only one discontinuous epitope was known based on 3D structures of two-chain antibody fragments with one-chain protein antigens [11].

Depending on the threshold values for parameters *R *and *S*, ElliPro predicted different number of epitopes in each protein; for an *R *of 6Å and *S *of 0.5, the average number of predicted epitopes in each protein analyzed was 4, with a variance from 2 to 8. For example, for *Plasmodium vivax *ookinete surface protein Pvs25 [PDB: 1Z3G, chain A], ElliPro predicted four epitopes with scores of 0.763, 0.701, 0.645, and 0.508, respectively (Fig. 2).

For each predicted epitope in each protein, we calculated the correctly (TP) and incorrectly predicted epitope residues (FN) and non-epitope residues, which were defined as all other protein residues (TN and FN). The statistical significance of a prediction, that is, the difference between observed and expected frequencies of an actual epitope/non-epitope residue in the predicted epitope/non-epitope, was determined using Fisher's exact test (right-tailed). The prediction was considered significant if the *P-value *was = 0.05. Then, for each prediction the following parameters were calculated:

*Sensitivity (recall or true positive rate (TPR)) = TP/(TP + FN) *– a proportion of correctly predicted epitope residues (TP) with respect to the total number of epitope residues (TP+FN).

*Specificity (or 1 – false positive rate (FPR)) = 1 - FP/(TN + FP) *– a proportion of correctly predicted non-epitope residues (TN) with respect to the total number of non-epitope residues (TN+FP).

*Positive predictive value (PPV) (precision) = TP/(TP + FP) *– a proportion of correctly predicted epitope residues (TP) with respect to the total number of predicted epitope residues (TP+FN).

*Accuracy (ACC) = (TP + TN)/(TP + FN + FP + TN) *– a proportion of correctly predicted epitope and non-epitope residues with respect to all residues.

*Area under the ROC Curve (AUC) *– area under a graph representing a dependency of TPR against FPR; that is, sensitivity against 1-specificity. The AUC gives the general performance of the method and is "equivalent to the probability that the classifier will rank a randomly chosen positive instance higher than a randomly chosen negative instance" [22].

For example, for the first predicted epitope in *Plasmodium vivax *ookinete surface protein Pvs25 [PDB:1Z3G, chain A] (Fig. 2), for an *R *of 6Å and *S *of 0.5, TP = 13, FP = 13, TN = 156, FN = 4, P-value = 5.55E-10, giving a sensitivity of 0.76, a specificity of 0.92, an accuracy of 0.91, and an AUC of 0.84. The results and detailed statistics of ElliPro performance for each epitope and other threshold values for *R *and *S *are provided in the supplementary materials [**see **Additional file 1].

**Figure 3:**
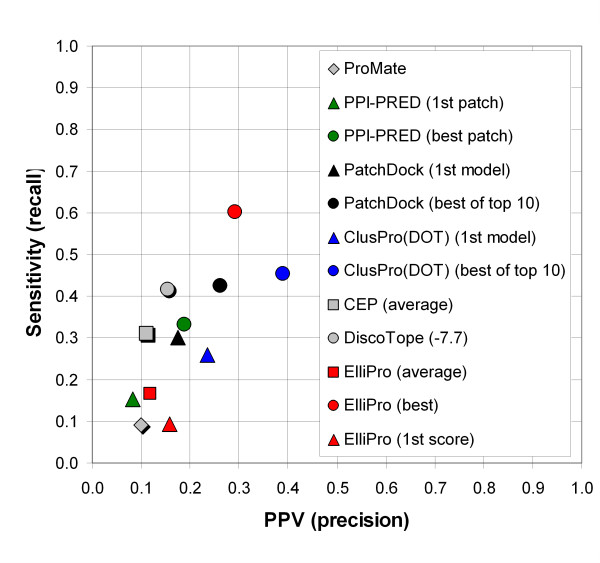
Overall ElliPro performance in comparison with other methods.

The statistics averaged over all epitopes and overall statistics calculated from FP, FN, TP, and TN values summarized for the whole pool of epitope and non-epitope residues are presented in Table 1 and Fig. 3. The results for the methods other than ElliPro have been obtained as described in [11]. ElliPro performed best, by AUC values, with the score *S *set at 0.7 and the distance *R *set at 6Å when the prediction with the highest score was considered for each protein and with the score *S *set at 0.5 and the distance *R *set at 6Å when the best by significance or average prediction was taken into account. Results are described using these thresholds (Table 1, Fig. 3); the results at other threshold values are provided in the supplementary materials [see Additional file 1].

**Table 1:** Overall performance of ElliPro in comparison with other methods^#^.

	**ElliPro (radius 6Å)**				**PPI-PRED**		**PatchDock**		**ClusPro (DOT)**			
	**Best prediction (score 0.5)**	**Average (score 0.5)**	**1st prediction (score 0.7)**	**ProMate (patch)**	**1st patch**	**best patch**	**1st model**	**best model of top 10**	**1st model**	**best model of top 10**	**CEP (average)**	**DiscoTope (-7.7)**
***Overall statistics***												
**sensitivity**	**0.601**	0.165	0.093	0.091	0.153	0.331	0.300	0.425	0.258	0.453	0.310	0.416
**1-specificity**	0.138	0.109	**0.047**	0.083	0.161	0.135	0.135	0.114	0.079	0.067	0.223	0.214
**precision**	0.291	0.119	0.158	0.101	0.083	0.188	0.175	0.262	0.235	**0.390**	0.110	0.155
**accuracy**	0.840	0.832	0.879	0.841	0.780	0.819	0.816	0.846	0.863	**0.892**	0.739	0.754
**AUC**	**0.732**	0.528	0.523	0.504	0.496	0.598	0.583	0.656	0.589	0.693	0.544	0.601
**P-value**	0.00E+00	1.37E-04	9.52E-06	2.74E-01	1.0E+00	7.8E-30	9.0E-23	0.0E+00	7.9E-34	0.0E+00	4.3E-06	4.1E-25
												
***Statistics averaged over epitopes***												
**sensitivity**	**0.58 ± 0.25**	0.17 ± 0.10	0.10 ± 0.20	0.09 ± 0.17	0.15 ± 0.24	0.34 ± 0.32	0.29 ± 0.26	0.42 ± 0.29	0.25 ± 0.31	0.46 ± 0.28	0.34 ± 0.28	0.43 ± 0.31
**1-specificity**	0.12 ± 0.10	0.11 ± 0.04	**0.05 ± 0.03**	0.08 ± 0.03	0.16 ± 0.07	0.14 ± 0.07	0.15 ± 0.06	0.13 ± 0.07	0.10 ± 0.07	0.08 ± 0.05	0.28 ± 0.20	0.22 ± 0.15
**precision**	**0.44 ± 0.22**	0.13 ± 0.06	0.16 ± 0.28	0.11 ± 0.20	0.10 ± 0.17	0.21 ± 0.24	0.19 ± 0.20	0.30 ± 0.25	0.25 ± 0.33	0.41 ± 0.29	0.11 ± 0.08	0.18 ± 0.12
**accuracy**	0.85 ± 0.08	0.82 ± 0.05	0.87 ± 0.05	0.83 ± 0.05	0.77 ± 0.07	0.81 ± 0.08	0.80 ± 0.08	0.83 ± 0.09	0.84 ± 0.09	**0.88 ± 0.07**	0.69 ± 0.17	0.74 ± 0.12
**AUC**	**0.73 ± 0.11**	0.53 ± 0.03	0.53 ± 0.10	0.51 ± 0.09	0.50 ± 0.13	0.60 ± 0.17	0.57 ± 0.14	0.64 ± 0.17	0.58 ± 0.17	0.69 ± 0.15	0.53 ± 0.08	0.60 ± 0.13

# – best prediction, patch, or model corresponds to the most significant (minimal P-value) of the predicted epitopes

ElliPro's top predictions, that are those with the highest scores, correlated poorly with the discontinuous epitopes known from 3D structures of antibody-protein complexes (Table 1, overall statistics, AUC = 0.523). DiscoTope and the first models from the docking methods performed better, giving AUC values above 0.6, whereas protein-protein binding site predicting methods, ProMate and PPI-PRED, performed worse. However, when the first predictions with the highest score were considered, ElliPro was the best among all the methods based on specificity (1-specificity = 0.047) and comparable with DiscoTope by precision (PPV = 0.158) (Table 1, overall statistics).

In a next set of metrics, we compared the performance between prediction methods when choosing the best hit within the top 10 predictions of each method. This approach takes into account that each antigen harbors multiple distinct binding sites for different antibodies. Therefore it is expected that the top predicted site is not necessarily recognized by the specific antibody used in the dataset. This comparison directly applies only to the docking methods DOT and PatchDock as well as ElliPro. For DiscoTope, only one epitope is predicted, while for CEP no ranking is available to identify the top 10 predictions.

The docking methods DOT and PatchDock have an intrinsic advantage in this comparison over ElliPro, because they use structures of both protein antigen and antibody from the same antibody-protein complex in order to predict binding sites. To our surprise, when the best significant prediction was considered for each protein, ElliPro nevertheless gave the highest AUC value of 0.732, the highest sensitivity of 0.601 and the second highest precision value of 0.29 among all the compared methods (Table 1; Fig. 3, red circle). The docking methods gave the AUC values of 0.693 for DOT and 0.656 for PatchDock, when also the best prediction of the top ten was considered (Table 1, overall statistics; Fig. 3). The average number of predicted epitopes for the analyzed proteins was four, with the rank of the best prediction at most fifth; for more than a half of proteins the rank was first or second, and the rank first, second, or third for more than 70% of all proteins [see Additional file 1].

ElliPro is based on simple concepts. First, regions protruding from the globular surface of the protein are more available for interaction with an antibody [9] and second those protrusions can be determined by treating the protein as a simple ellipsoid [20]. Obviously, this is not always the case, especially for multi-domain or large single-domain proteins. However, no correlation between the protein size, which varied from 51 to 429 residues with an average value of 171, or number of domains (8 proteins among the 39 analyzed contained more than one domain) and ElliPro performance was found (data not shown).

## Conclusion

ElliPro is a web-based tool for the prediction of antibody epitopes in protein antigens of a given sequence or structure. It implements a previously developed method that represents the protein structure as an ellipsoid and calculates protrusion indexes for protein residues outside of the ellipsoid. ElliPro was tested on a benchmark dataset of discontinuous epitopes inferred from 3D structures of antibody-protein complexes. In comparison with six other structure-based methods that can be used for epitope prediction, ElliPro performed the best (AUC value of 0.732) when the most significant prediction was considered for each protein. Since the rank of the best prediction was at most three in more than 70% of proteins and never exceeded five, ElliPro is considered a potentially useful research tool for identifying antibody epitopes in protein antigens.

While ElliPro was tested on antibody-protein binding sites, it might be interesting to test it on other protein-protein interactions since it implements a method that is based on geometrical properties of protein structure and does not require training.

Comparison with DiscoTope, which is based on training and utilizes epitope features such as amino acid propensities, residue solvent accessibility, spatial distribution, and inter-molecular contacts, suggests that further research on antibody epitopes which considers more features that discriminate epitopes from non-epitopes may improve the prediction of antibody epitopes.

## Availability and requirements

• **Project name: **ElliPro

• **Project home page: **

• **Operating system(s): **Platform independent

• **Programming language: **Java

• **Other requirements: **None

• **License: **None

• **Any restrictions to use by non-academics: **None

## Abbreviations

PI: protrusion index; TP: true positives; FP: false positives; TN: true negatives, FN: false negatives; ROC: Receiver Operating Characteristics; AUC: area under the ROC curve.

## Authors' contributions

HHB conceived, designed and programmed the tool. JVP tested the tool and wrote the manuscript. WL and NF participated in programming the tool. PEB, BP and AS contributed to writing the manuscript. All authors have read and approved the final version of the manuscript.

## Supplementary Material

Additional file 1 **The detailed statistics on the prediction results for 39 epitopes analyzed**. This table provides additional information that complements the Table 1 and Figure 3. Click here for file
